# Nanomaterials in Drug Delivery: Leveraging Artificial Intelligence and Big Data for Predictive Design

**DOI:** 10.3390/ijms262211121

**Published:** 2025-11-17

**Authors:** Youngji Han, Dong Hyun Kim, Seung Pil Pack

**Affiliations:** 1 Bio-Medical Research Institute, Kyungpook National University Hospital, Daegu 41940, Republic of Korea; youngjihan@knu.ac.kr; 2 Department of Biotechnology and Bioinformatics, Korea University, Sejong 30019, Republic of Korea; jklehdgus@korea.ac.kr; 3 Biological Clock-Based Anti-Aging Convergence RLRC, Korea University, Sejong 30019, Republic of Korea

**Keywords:** nanomaterials, drug delivery, artificial intelligence, machine learning, deep learning, nanoinformatics, predictive modeling, precision medicine

## Abstract

Nanomaterials have revolutionized drug delivery by enabling precise control over solubility, stability, circulation time, and targeted release, yet translation from bench to bedside remains challenging due to complex synthesis, unpredictable biological interactions, and regulatory hurdles. Recent advances in artificial intelligence (AI) and big data analytics offer powerful solutions to these bottlenecks by integrating multidimensional datasets—encompassing physicochemical characterization, pharmacokinetics, omics profiles, and preclinical outcomes—to generate predictive models for rational nanocarrier design. Machine learning and deep learning approaches enable the prediction of key parameters such as particle size, drug loading efficiency, and biodistribution, while generative algorithms explore novel chemistries and architectures optimized for specific clinical applications. Nanoinformatics platforms and large-scale data repositories further enhance reproducibility and cross-study comparisons, supporting regulatory science and accelerating clinical translation. This review provides a comprehensive overview of nanomaterial-based drug delivery systems, highlights AI-driven strategies for predictive modeling and optimization, and discusses translational and regulatory perspectives. By bridging nanotechnology, computational modeling, and precision medicine, AI-assisted nanomaterial design has the potential to transform drug delivery into a more efficient, reproducible, and patient-centered discipline.

## 1. Introduction

Nanomaterials have emerged as transformative platforms in modern drug delivery, offering unique opportunities to overcome the limitations of conventional therapeutics [1,2]. Traditional drug formulations often suffer from poor solubility, low stability, rapid clearance, and lack of tissue specificity, all of which reduce therapeutic efficacy and increase systemic side effects [3]. In contrast, nanomaterials can be engineered with finely tunable physicochemical properties such as particle size, shape, surface charge, porosity, and surface functionalization [4,5]. These features enable not only improved solubility and circulation time but also controlled drug release, enhanced cellular uptake, and precise delivery to diseased tissues or organs [6]. For instance, liposomes, polymeric nanoparticles, dendrimers, and inorganic nanostructures have been successfully tested for applications in cancer therapy, gene delivery, and treatment of metabolic and neurodegenerative disorders [7,8]. Such progress highlights the enormous potential of nanocarriers to transform patient care by bridging the gap between pharmacological innovation and clinical need [9].

Despite these advances, the path from laboratory innovation to clinical translation remains fraught with challenges. The synthesis of nanomaterials often involves complex chemical procedures that can introduce variability in particle size distribution, stability, and reproducibility [10]. Furthermore, the biological performance of nanocarriers depends heavily on their interactions with proteins, immune cells, and other components of the biological milieu, which are difficult to predict using conventional experimental approaches [11]. As a result, many promising nanomaterials demonstrate efficacy in preclinical models but fail in clinical trials due to unforeseen toxicity, poor biodistribution, or lack of therapeutic benefit [12]. Addressing this translational bottleneck requires a more systematic and predictive approach to nanomaterial design and evaluation, one that can integrate multidimensional datasets encompassing chemical synthesis, physicochemical characterization, pharmacokinetics, and biological responses [13].

Artificial intelligence (AI) and big data analytics are increasingly recognized as powerful tools to overcome these barriers. Machine learning algorithms and computational modeling can analyze vast and heterogeneous datasets, uncover hidden relationships, and generate predictive models that guide the rational design of nanocarriers [14,15]. For example, supervised learning methods can predict drug-nanoparticle interactions and optimize release kinetics, whereas unsupervised learning can cluster nanomaterials based on their structural or functional similarities [16]. More advanced approaches, such as deep learning and neural networks, have demonstrated the ability to model nonlinear biological interactions and forecast in vivo outcomes with higher accuracy than traditional statistical techniques [17]. In parallel, emerging nanoinformatics databases provide standardized repositories of experimental data, enabling cross-study comparisons and facilitating reproducibility [18]. Together, these AI-driven strategies promise to accelerate the discovery of clinically viable nanomaterials, reduce the time and cost associated with experimental testing, and ultimately improve patient outcomes [19].

This review provides a comprehensive overview of how AI is being integrated into the development of nanomaterial-based drug delivery systems. We summarize recent advances in predictive modeling, highlight case studies where AI has successfully forecasted the behavior of nanomaterials in biological environments, and discuss the synergistic role of big data platforms in enabling these advances [20]. Finally, we address the remaining challenges, such as data quality, model interpretability, and regulatory considerations, while outlining future directions for the field. By bridging nanotechnology with AI, we aim to illustrate a paradigm shift that could transform the design, optimization, and clinical translation of next-generation drug delivery systems.

## 2. Nanomaterials in Drug Delivery

**Figure 1:**
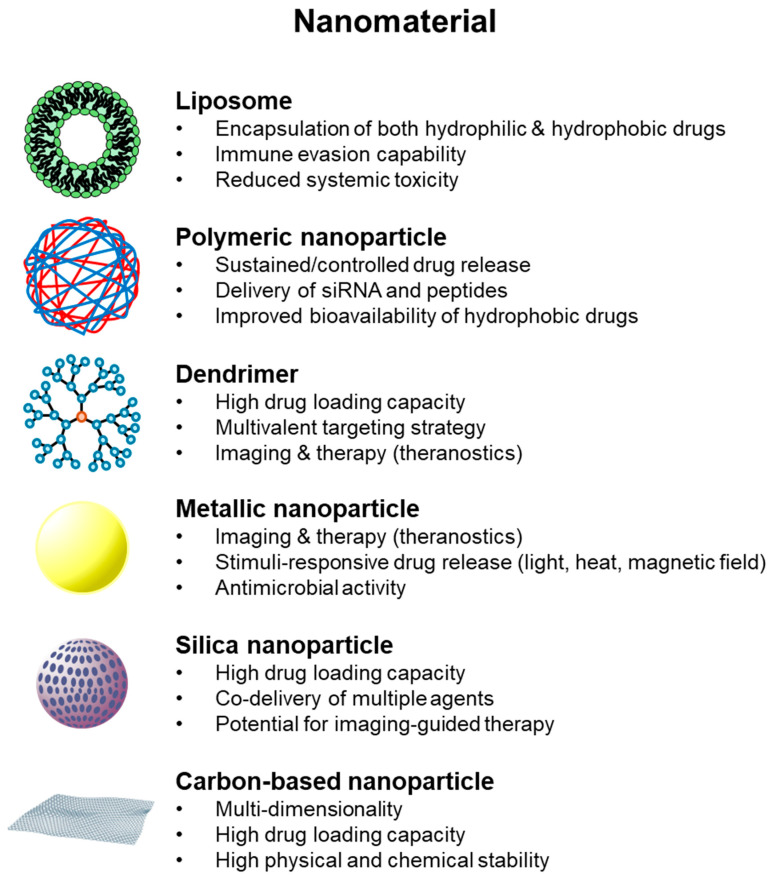
Overview of nanomaterial-based platforms for drug delivery.

A wide spectrum of nanomaterials has been developed and tested for drug delivery applications, each offering distinct physicochemical features and therapeutic advantages [6,21]. Among the most extensively studied platforms are liposomes, polymeric nanoparticles, micelles, dendrimers, metallic nanostructures, and silica-based carriers, as shown in Table 1 and Figure 1 [5,22,23]. These systems collectively enable prolonged circulation, site-specific accumulation, controlled drug release, and in many cases, the possibility of multifunctionality such as simultaneous imaging and therapy [7]. Moreover, their tunable characteristics allow researchers to tailor nanocarriers to specific clinical contexts, ranging from oncology to infectious diseases and neurodegenerative disorders [24,25].

### 2.1. Liposome

Liposomes represent one of the earliest and most clinically successful nanocarrier systems. Their biocompatible lipid bilayer allows for the encapsulation of both hydrophilic and hydrophobic compounds, while surface modification with polyethylene glycol (PEGylation) prolongs systemic circulation and reduces immune clearance by hindering opsonin binding and decreasing uptake by the mononuclear phagocyte system (MPS) [39,40]. Several liposomal formulations, such as PEGylated doxorubicin-liposomes (e.g., Doxil^®^, LipoDox^®^), non-PEGylated variants (e.g., Myocet^®^), and liposomal combinations like Vyxeos^®^ (cytarabine + daunorubicin) have already reached clinical approval for various cancers, highlighting their translational relevance [41,42]. Post-approval studies also show improved pharmacokinetics, reduced cardiotoxicity, and enhanced tumor accumulation via the enhanced permeability and retention effect (EPR) effect for these liposomal drugs compared to their conventional counterparts [43].

### 2.2. Polymeric Nanoparticle

Polymeric nanoparticles, typically composed of biodegradable polymers like poly(lactic-co-glycolic acid) (PLGA) or polycaprolactone, provide controlled and sustained drug release over extended periods. Their tunability in terms of particle size, degradation rate, and surface functionalization allows for precise pharmacokinetic control, reduced systemic toxicity, and targeted tissue accumulation [44]. Peptide- and siRNA-loaded PLGA systems have demonstrated tri-phasic release profiles, enhanced intracellular uptake, and improved therapeutic outcomes in cancer and gene-silencing models [45,46]. Recent studies have shown that PLGA-based nanoparticles can efficiently encapsulate and protect siRNA from nuclease degradation, enhance endosomal escape, and achieve potent gene silencing in vivo [47].

Polymeric micelles, formed by the self-assembly of amphiphilic block copolymers, are particularly suited for improving the solubility and oral bioavailability of poorly water-soluble drugs. Several micelle-based formulations have already advanced to clinical approval or late-stage trials. For instance, Genexol-PM^®^ (paclitaxel-loaded PEG-PLA micelles) is approved in South Korea and other countries for breast cancer, showing higher maximum tolerated doses and reduced hypersensitivity compared to Cremophor-based paclitaxel [29,48]. Other candidates such as NK105 and SP1049C have demonstrated improved tumor accumulation and reduced systemic toxicity in Phase II/III trials [49,50]. Together, these findings underscore the translational relevance of polymeric nanoparticle and micelle platforms in precision drug delivery.

### 2.3. Dendrimer

Dendrimers offer a highly branched, tree-like architecture with precise molecular weight and monodisperse surface functionalities. This well-defined structure allows for exceptionally high drug-loading capacity, multivalent ligand presentation, and tunable surface chemistry, enabling selective targeting of receptors or biomolecules [51,52]. Beyond small-molecule delivery, dendrimers have been explored for gene therapy, siRNA delivery, and imaging applications, often showing improved cellular uptake and reduced off-target toxicity compared to linear polymers [32]. Poly(amidoamine) (PAMAM) dendrimers are the most extensively studied, with early-phase clinical trials evaluating their use in anti-HIV microbicides (e.g., VivaGel^®^) and targeted anticancer drug delivery [53,54]. These attributes make dendrimers versatile nanoplatforms for both therapeutic and diagnostic (theranostic) purposes.

### 2.4. Metallic Nanoparticle

Inorganic nanomaterials provide an additional dimension of functionality in nanomedicine. Metallic nanoparticles (NPs), including gold (AuNPs), silver (AgNPs), and iron oxide nanoparticles (IONPs), have been extensively investigated for drug delivery, as well as for theranostic applications that combine therapeutic payload delivery with real-time imaging [55,56]. Their unique optical (surface plasmon resonance), magnetic, and electronic properties allow externally triggered drug release using stimuli such as near-infrared light for photothermal therapy, alternating magnetic fields for magnetothermal therapy, or ultrasound [57,58].

Gold nanoparticles have been widely studied for photothermal ablation of tumors, and early-phase clinical trials have demonstrated promising efficacy and safety profiles [59]. Iron oxide nanoparticles, which are already approved as MRI contrast agents (e.g., Feridex^®^, Ferumoxytol^®^), are being repurposed as drug carriers and immune modulators [31,59]. Biosilica-supported silver nanoparticles, such as those synthesized using green chemistry approaches, have exhibited potent antibacterial activity and excellent reusability, highlighting their potential for combined antimicrobial and drug-delivery applications [23]. Collectively, these findings demonstrate that metallic nanoparticles are highly promising candidates for stimuli-responsive, image-guided drug delivery systems.

### 2.5. Silica-Based Nanoparticle

Silica-based nanoparticles, including mesoporous silica nanoparticles (MSNs), are characterized by their high surface area, tunable pore size, and easily modifiable surface chemistry [60,61]. These properties enable highly efficient drug loading, protection of cargo from premature degradation, and precisely controlled release profiles. Functionalization with targeting ligands, pH-responsive linkers, or polymer coatings can further enhance tumor-specific accumulation and reduce systemic toxicity [62].

MSNs have been widely investigated for co-delivery of multiple therapeutic agents, such as chemotherapeutic drugs and siRNA, to achieve synergistic treatment effects [63]. In preclinical studies, MSN-based formulations have demonstrated improved therapeutic indices, reduced off-target effects, and the ability to bypass multidrug resistance [64]. Recent advances also explore MSNs for imaging-guided therapy, where fluorescent or magnetic labels are incorporated to track biodistribution in real time [65]. These attributes make silica-based nanoparticles highly versatile platforms for next-generation combination therapies and theranostic applications.

### 2.6. Carbon-Based Nanoparticle

Carbon-based nanoparticles are utilized as drug carriers in various structural forms due to the unique bonding characteristics of carbon atoms and nanoscale effects. Specifically, these nanomaterials are classified based on the dimensionality of their nanostructures [66]. Zero-dimensional structures include carbon dots and fullerenes, one-dimensional structures include carbon nanotubes, and two-dimensional structures include graphene. These diverse structures provide a large surface area, which is advantageous for attaching drugs and functionalizing surfaces [67].

Carbon-based nanomaterials are being extensively researched in a variety of biomedical fields, including drug and gene delivery, bioimaging, biosensing, photothermal and photodynamic therapy, and tissue engineering [68]. For example, in the context of cancer treatment, amino acid-mimicking carbon dots with guanidinium functionalization were synthesised to encapsulate siRNA [37]. Lee et al. used nanodiamonds, a type of carbon-based nanomaterial, to treat apical lesions. The results demonstrated efficacy in terms of healing, pain reduction and preventing reinfection, thus setting a precedent for human application [38].

## 3. Role of Artificial Intelligence in Nanomaterial Design

AI has emerged as a transformative force in the rational design and development of nanomaterials for drug delivery, marking a shift from conventional trial-and-error experimentation toward predictive, data-driven optimization [69,70]. This paradigm shift is particularly critical given the vast design space of nanocarriers, where subtle variations in size, morphology, surface chemistry, and surface functionalization can produce dramatically different pharmacokinetic and pharmacodynamic outcomes [71].

By integrating AI methodologies—including machine learning, deep learning, and computational modeling—researchers can systematically explore this multidimensional landscape, uncover non-obvious correlations, and prioritize candidate nanostructures with optimal therapeutic profiles [72]. Such approaches not only shorten experimental cycles and reduce cost but also improve translational predictability, bridging the gap between preclinical discovery and clinical implementation [20].

**Figure 2:**
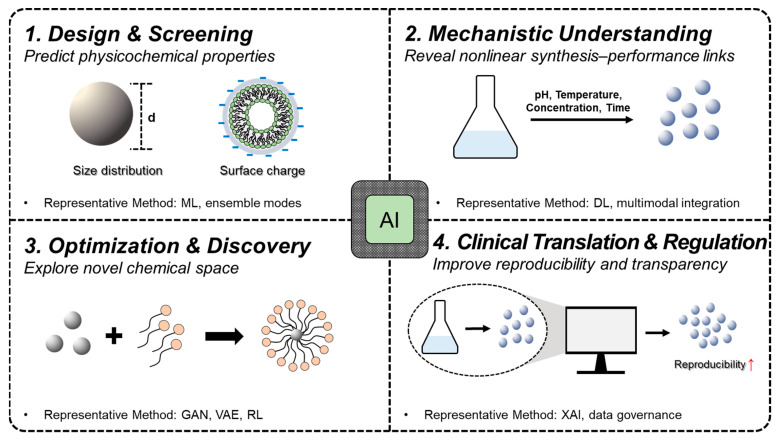
AI-driven strategies supporting nanomaterial design, optimization, and clinical translation.

AI facilitates innovation in four interconnected domains: Design & Screening—prediction of physicochemical properties such as size distribution and surface charge using machine learning (ML) and ensemble models; Mechanistic Understanding—elucidation of nonlinear synthesis–performance relationships (e.g., pH, temperature, concentration, time) through deep learning (DL) and multimodal data integration; Optimization & Discovery—exploration of novel chemical spaces using generative models such as generative adversarial networks (GANs), variational autoencoders (VAEs), and reinforcement learning (RL); Clinical Translation & Regulation—enhancement of reproducibility and transparency in preclinical and regulatory settings through explainable AI (XAI) and data governance frameworks (Figure 2, Table 2).

### 3.1. Machine Learning

Machine learning (ML) algorithms, including support vector machines (SVM), random forests (RF), gradient boosting methods (e.g., XGBoost), and ensemble learning frameworks, have been successfully applied to predict key physicochemical and biological parameters of nanocarriers [73,74,75]. These parameters include particle size distribution, zeta potential, colloidal stability, drug encapsulation efficiency, and in vitro cytotoxicity profiles. By learning from large experimental datasets, ML models can map complex, nonlinear relationships between synthesis conditions and resulting nanomaterial properties [76].

Such predictive modeling not only reduces the number of costly and time-consuming experimental iterations required but also yields mechanistic insights into how formulation variables influence downstream biological performance. This allows researchers to rapidly prioritize optimal formulations before entering preclinical and clinical testing, thereby accelerating translational pipelines [77].

### 3.2. Deep Learning

Deep learning approaches have further expanded these capabilities by enabling the modeling of complex, nonlinear relationships that govern nanomaterial behavior in biological systems. Convolutional neural networks (CNNs) and recurrent neural networks (RNNs) can process high-dimensional datasets—such as TEM/imaging-derived nanoparticle morphology or time-series pharmacokinetic data—and improve accuracy in forecasting how design parameters (polymer composition, ligand density, surface charge, etc.) map to biodistribution, cellular uptake, and therapeutic efficacy [78,79,80,81]. Importantly, deep learning frameworks can integrate multimodal data (chemical descriptors, omics readouts, bioassay results, and image features), offering a holistic perspective that traditional regression models cannot achieve [79,80,81,82,83]. For example, DNN-augmented PBPK pipelines have already been shown to learn nonlinear structure–biodistribution links for tumor delivery, while CNNs trained on microscopy images automatically extract morphology descriptors that correlate with downstream biological performance [81,82,83].

### 3.3. Generative Design Strategies

Artificial intelligence is increasingly applied to generative design, which seeks to create new nanocarrier candidates rather than merely optimizing existing ones. Variational autoencoders (VAEs) and generative adversarial networks (GANs) learn latent representations of nanocarrier features and propose designs with optimized size, charge, and release profiles [84,85,86,87]. Reinforcement learning (RL) further frames this as a decision-making process to maximize therapeutic efficacy while minimizing off-target effects [88].

These approaches, initially developed for materials discovery, are now applied to nanomedicine. GAN-based models have been used to predict intratumoral nanoparticle distribution, and VAEs or diffusion models are being adapted to explore complex lipid and polymer design spaces. Protein design tools such as RFdiffusion and AlphaFold enable the creation of receptor-specific ligands that can be displayed on nanocarriers to improve targeting [87,88,89,90].

Combined with multitask predictors in an active learning loop, these generative models reduce costly experimentation and accelerate the identification of candidates with desirable biodistribution and safety profiles [87,89,91]. Although fully validated end-to-end studies are still emerging, evidence suggests that generative strategies expand the accessible design space and support faster clinical translation of nanomedicines [89,90,91,92].

### 3.4. Critical Perspective on AI-Driven Modeling Approaches

While AI techniques have significantly advanced predictive modeling in nanomedicine, it is crucial to evaluate their assumptions and limitations critically [93]. Supervised learning approaches offer high accuracy when trained on large, well-annotated datasets; however, they struggle with generalization when data diversity is limited or bias exists in the training distribution [93]. In contrast, unsupervised and self-supervised methods can discover hidden patterns in unlabeled data, but often lack quantitative predictive power for clinical endpoints [94,95]. Deep learning models capture nonlinear relationships between nanomaterial structure and biological response, surpassing traditional regression or QSAR methods in accuracy; however, their “black-box” nature reduces interpretability and hampers regulatory trust [96]. Mechanistic modeling, in contrast, retains explainability and causal consistency but relies on simplified biological assumptions and is computationally demanding for complex systems. The future of AI-assisted nanomedicine will likely depend on hybrid frameworks that combine mechanistic and data-driven modeling to achieve both predictive power and biological interpretability [97]. Such integrative approaches can bridge the gap between in silico predictions and in vivo performance, enhancing translational reliability while reducing experimental burden and ethical concerns associated with animal testing.

## 4. Big Data and Nanoinformatics

### 4.1. Emergence of Nanoinformatics

**Figure 3:**
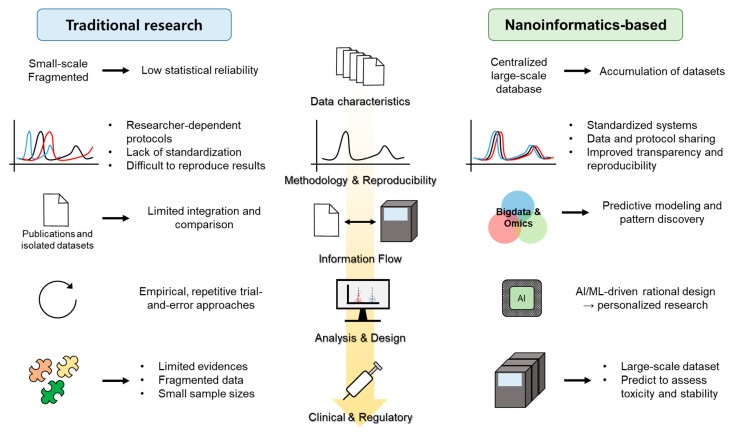
Comparison between traditional nanomedicine research and nanoinformatics-based big data approaches.

The rapid expansion of nanoinformatics platforms and big data resources has profoundly reshaped how nanomaterials are characterized, analyzed, and applied in drug delivery research [98,99] Traditional nanomedicine development has relied on isolated experimental studies, often limited by small sample sizes, inconsistent methodologies, and difficulties in reproducing results across laboratories. In contrast, the emergence of centralized databases and standardized reporting systems now enables researchers to access and integrate vast amounts of structured information linking nanomaterial composition, physicochemical attributes, and biological outcomes [100]. This systematic approach lays the groundwork for predictive modeling, comparative analyses, and evidence-based design of next-generation nanocarriers (Figure 3).

### 4.2. Major Databases and Repositories

Several key repositories exemplify the growing impact of nanoinformatics. The Nanomaterial Registry aggregates data on thousands of nanomaterials, cataloging details such as particle size, shape, surface charge, coating chemistry, and biological responses [101]. Similarly, caNanoLab, developed by the National Cancer Institute, provides a platform for annotating nanomaterial protocols, characterizations, and experimental results, thereby promoting reproducibility and transparency in nanomedicine research [102]. In addition, PubChem and other chemical databases extend this framework by linking nanomaterial descriptors to broader molecular datasets, facilitating cross-domain comparisons and hypothesis generation [103].

### 4.3. Integration with Big Data Analytics

Beyond descriptive cataloging, the integration of big data analytics has elevated nanoinformatics into a predictive and translational discipline [13]. By applying advanced machine learning algorithms to these repositories, researchers can uncover hidden patterns in how nanomaterials interact with biological systems [104]. Importantly, big data approaches allow the fusion of heterogeneous datasets, including preclinical toxicology studies, clinical trial results, and multi-omics profiles [105]. This integration creates unique opportunities for personalized nanomedicine, where patient-specific genomic, proteomic, or metabolomic information can be matched with nanoparticle libraries to predict optimal drug–nanocarrier combinations [106].

### 4.4. Regulatory Science and Translational Impact

Big data-driven frameworks also play a pivotal role in regulatory science and safety evaluation [107]. Regulatory agencies increasingly require systematic evidence for the safety and efficacy of nanomaterials, and predictive models based on large-scale datasets can support this process by forecasting toxicity risks, biodistribution patterns, and long-term stability [108]. Moreover, integrating explainable AI techniques into nanoinformatics platforms can improve transparency, facilitating acceptance among regulators, clinicians, and patients alike [109].

## 5. Predictive Modeling and Optimization

### 5.1. Molecular and Computational Modeling

Molecular and computational modeling provide a foundation for understanding nanocarrier behavior in biological systems before entering labor-intensive experiments. Molecular dynamics (MD) simulations offer atomistic insights into nanoparticle interactions with lipid bilayers, proteins, and nucleic acids, enabling predictions of membrane penetration, endocytosis pathways, and drug release mechanisms [110,111]. Quantum mechanical (QM) calculations, though computationally intensive, deliver precise information about electronic structures, binding energies, and surface reactivity of nanomaterials [112]. Together, MD and QM simulations create high-resolution datasets that guide rational nanocarrier design and de-risk early-stage development.

### 5.2. Machine Learning-Enhanced Prediction

Machine learning has recently been applied to predict nanoparticle biodistribution and clearance with high accuracy by leveraging large experimental datasets of physicochemical properties and in vivo pharmacokinetics. For example, Wu et al. developed a generalized physiologically based pharmacokinetic (PBPK) model integrated with machine learning to predict the organ-level distribution of diverse nanoparticles, including gold, silica, and iron oxide, across multiple animal models, demonstrating strong agreement with experimental data and improved extrapolation to humans [92]. These approaches significantly reduce the need for extensive in vivo screening and accelerate the rational design of nanocarriers for clinical translation. Complementary reviews highlight that such data-driven frameworks can be combined with QSAR and multi-omics datasets to enable predictive, patient-specific nanomedicine design and streamline regulatory evaluation [74].

### 5.3. Formulation and Route Optimization

AI-driven models have also been applied to optimize formulation variables and delivery routes. By analyzing datasets comparing oral, intravenous, subcutaneous, and inhalation pathways, ML algorithms can identify the most effective strategy for a given nanocarrier–drug combination [113]. These insights inform the design of nanoparticles with enhanced oral bioavailability, stability under gastrointestinal conditions, or aerodynamic properties suitable for pulmonary delivery, ultimately improving therapeutic outcomes and patient adherence.

### 5.4. Toward Precision and Personalized Nanomedicine

The next frontier for predictive modeling lies in precision medicine. Integrating patient-specific genomic, epigenomic, and metabolomic profiles into AI-guided models enables the design of nanocarriers tailored to individual patients [114]. Such personalization is particularly valuable in oncology, where tumor heterogeneity limits one-size-fits-all approaches, and in neurodegenerative diseases, where crossing the blood–brain barrier remains a challenge. Personalized modeling holds promise for developing nanomedicines that maximize efficacy while minimizing off-target toxicity.

### 5.5. Translational and Regulatory Perspectives

The integration of AI into nanomaterial design has emerged as a powerful driver of translational research. By reducing experimental workload, prioritizing high-probability candidates, and predicting physicochemical and biological behavior with high accuracy, AI-driven approaches lower development costs, shorten timelines, and increase the likelihood that preclinical results will translate effectively to human trials [73,115,116]. Recent studies demonstrate that ML and DL models can predict nanoparticle toxicity, biodistribution, and pharmacokinetics with superior accuracy compared to conventional screening methods, thereby accelerating candidate selection and de-risking clinical entry [74].

Equally important is the rapid growth of explainable AI (XAI) approaches, which enhance model interpretability and provide transparent reasoning for predictions. These advances facilitate regulatory acceptance, support reproducibility, and foster greater trust between researchers and clinicians [20]. Finally, the implementation of rigorous data governance, standardized reporting, and the integration of high-quality, representative datasets are essential to ensure the reproducibility and generalizability of AI-assisted nanomedicine pipelines [100].

**Figure 4:**
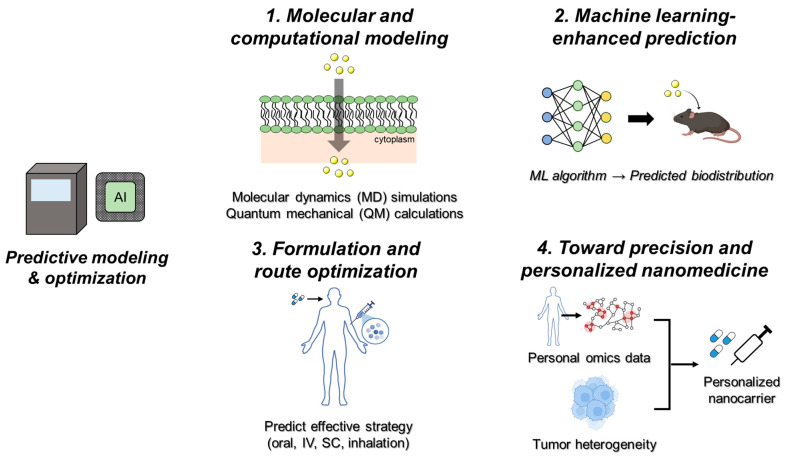
Predictive modeling and optimization workflow for nanomaterial-based drug delivery using AI and big data [74,112,113,114].

Together, these developments underscore the transformative role of AI in nanomaterial-based drug delivery. By combining predictive accuracy with creative generative design, AI enables rational, efficient, and clinically relevant nanocarrier development that moves beyond the empirical trial-and-error paradigm. By aligning design, mechanistic understanding, optimization, and clinical translation with AI-driven methodologies, this framework highlights how predictive modeling, generative design, and explainable AI collectively accelerate discovery, enhance reproducibility, and improve the likelihood of successful clinical implementation (Figure 4).

Recent advances have demonstrated that AI-assisted nanocarrier design can directly impact clinical and regulatory outcomes. For example, Pfizer and BioNTech utilized a machine learning-guided lipid nanoparticle (LNP) formulation platform to optimize the physicochemical stability and immunogenicity of mRNA vaccines, contributing to the accelerated development of the first FDA-authorized mRNA-based nanomedicine [117]. In parallel, the U.S. FDA’s National Center for Toxicological Research (NCTR) and the EU-funded NanoSolveIT project have developed predictive nanotoxicology models capable of estimating biodistribution and cytotoxicity across multiple nanoparticle classes [118,119]. These initiatives represent a crucial step toward data-driven regulatory science, where AI models inform safety evaluations and risk assessments. Collectively, they exemplify how AI-based modeling and optimization are evolving from experimental research tools into validated components of clinical translation and policy decision-making.

## 6. Opportunities and Challenges

The integration of AI and big data analytics into nanomaterials research offers unprecedented opportunities to accelerate innovation and translation in drug delivery. By leveraging predictive modeling and data-driven optimization, researchers can significantly shorten development timelines, reduce experimental costs, and identify high-value candidates more efficiently than traditional trial-and-error approaches [114,120]. These methods enable rational and reproducible nanocarrier design, thereby improving the likelihood of clinical success and aligning with the broader vision of precision medicine, where therapeutic strategies are tailored to the molecular, genetic, and physiological profiles of individual patients [5]. Consequently, AI-assisted nanomedicine has the potential to not only improve drug efficacy and safety but also transform healthcare into a more patient-centered paradigm.

Despite these advantages, several persistent challenges remain. A major limitation is data scarcity and heterogeneity. Many nanomaterial studies are performed with small sample sizes, inconsistent characterization protocols, and limited in vivo validation, which restricts the robustness and generalizability of predictive models [100]. Cross-study integration is further hindered by the lack of standardized ontologies and reporting frameworks, limiting the ability to perform meta-analyses and comparative modeling.

Another critical issue is the interpretability of AI models. While deep learning approaches can achieve remarkable predictive accuracy, their “black box” nature raises concerns regarding transparency and trustworthiness. Clinicians, regulators, and patients alike require a clear understanding of how models generate predictions, particularly in high-stakes applications such as drug safety and efficacy. The development of explainable AI (XAI) methods is therefore essential to bridge the gap between computational predictions and practical decision-making. For instance, Holzinger et al. distinguish between explainability and causability in medical AI, arguing that systems must support human-understandable explanations, not just algorithmic transparency [109].

Progress will also depend heavily on collaborative data sharing and standardized data infrastructures. Initiatives such as the Nanomaterial Registry and caNanoLab provide valuable platforms for structured data curation, but broader adoption and harmonization of reporting standards are needed to ensure reproducibility [121]. Open access to experimental protocols, toxicology data, and clinical trial outcomes will be essential for building the diverse, high-quality datasets required for robust predictive modeling.

The integration of data-driven strategies into regulatory decision-making is increasingly shaping the future of nanomedicine development. Regulatory agencies such as the FDA and EMA are emphasizing systematic evidence collection, standardized characterization, and predictive modeling to streamline approval pathways and improve safety evaluation [122]. Centralized nanomaterial registries and computational frameworks support cross-study comparison and meta-analysis, enabling regulators to assess risk–benefit profiles more consistently [74]. Recent efforts highlight the value of machine learning and computational modeling to predict toxicity, biodistribution, and immunogenicity before entering clinical trials [123]. By aligning with data-rich, evidence-based approaches, these strategies accelerate translation while maintaining rigorous safety standards, paving the way for reliable and reproducible nanomedicine development.

Taken together, these opportunities and challenges underscore a pivotal moment for the field. Addressing these limitations through interdisciplinary collaboration, improved data infrastructures, and transparent AI methodologies will unlock the full potential of AI-assisted nanomaterials and pave the way for safer, more effective, and personalized drug delivery systems.

While the integration of artificial intelligence and big data into nanomaterials research offers transformative potential, several limitations should be acknowledged. Current predictive models are often constrained by the quality, quantity, and consistency of available data, with many studies relying on small-scale or heterogeneous datasets that limit generalizability [100]. The lack of standardized protocols for nanomaterial characterization and biological testing further hampers cross-study comparability, making it difficult to establish robust training datasets for machine learning [102]. Moreover, the interpretability of deep learning models remains a challenge, raising concerns about transparency and clinical applicability [69,124]. Beyond technical challenges, ethical and regulatory aspects are becoming increasingly central to the deployment of AI-driven nanomedicine [74,125]. Algorithmic bias arising from unbalanced or non-representative datasets can lead to inequitable therapeutic recommendations or inaccurate toxicity predictions across populations [126]. Ensuring data privacy and patient safety is essential when integrating omics and clinical information into predictive frameworks. Transparent reporting and the development of explainable AI (XAI) are indispensable for maintaining accountability and trust between regulators, clinicians, and patients. Additionally, it is essential to establish clear governance structures and ethical guidelines for AI-assisted decision-making, including model ownership, validation, and post-approval monitoring. Such frameworks will be crucial for responsible translation and for maintaining public confidence in AI-enabled nanotherapeutics [74]. Another limitation lies in the gap between computational predictions and real-world performance, as in vitro and in silico results may not fully capture the complexity of in vivo biological systems [5,12]. Finally, ethical and regulatory uncertainties surrounding AI-driven design approaches present additional barriers to clinical translation [127,128,129]. Addressing these limitations will be essential to ensure that AI-assisted nanomaterial design achieves its promise of safe, reproducible, and patient-centered drug delivery solutions.

## 7. Conclusions and Future Perspectives

Nanomaterials remain at the forefront of drug delivery innovation, offering unprecedented opportunities to enhance therapeutic precision, improve bioavailability, and enable multifunctional treatment strategies. Yet, the complexity of their synthesis, characterization, and biological interactions highlights the need for approaches that transcend conventional empirical experimentation. AI and big data analytics have emerged as transformative tools, providing the computational capacity and predictive power required for rational nanocarrier design, optimization, and clinical translation.

Future research must focus on several key directions to realize the full potential of AI-assisted nanomedicine. The integration of multi-omics datasets—including genomics, transcriptomics, proteomics, metabolomics, and microbiome profiles—will be essential for understanding patient-specific responses to nanomaterials and advancing the paradigm of precision nanomedicine. Such integration can inform predictive models that anticipate therapeutic efficacy and minimize adverse effects across heterogeneous populations.

Developing interpretable and transparent AI models is equally critical for building trust between researchers, clinicians, and regulators. Incorporating explainability, causal inference, and mechanistic modeling into AI frameworks will bridge the gap between computational predictions and clinical decision-making.

The establishment of standardized, interoperable databases will form the foundation for reproducible research and cross-laboratory comparability. Harmonization of experimental protocols, data annotation, and reporting standards will facilitate robust machine learning applications and strengthen international collaboration. These advances will also support regulatory science by providing validated predictive models that streamline approval processes and ensure safety and efficacy.

Over the next decade, several emerging trends are expected to shape the evolution of AI-assisted nanomedicine. Federated learning will enable secure and privacy-preserving model training across multiple institutions, thereby enhancing data diversity and model robustness. Quantum computing-based nanodesign will allow the rapid simulation of complex nanoscale interactions that are currently beyond the reach of classical computing. In parallel, human–AI collaborative frameworks, in which researchers interact directly with generative models, will accelerate hypothesis generation, nanocarrier optimization, and regulatory documentation. Collectively, these developments will usher in a new era of transparent, reproducible, and ethically responsible nanomedicine, connecting computational innovation with clinical translation.

Taken together, the convergence of AI, big data, and nanomaterial science represents a paradigm shift in drug delivery research. By overcoming current limitations and embracing these future opportunities, AI-assisted nanomaterial design can accelerate the translation of laboratory discoveries into safe, effective, and patient-centered therapies, ultimately redefining the landscape of precision medicine.

## Figures and Tables

**Table 1:** Representative nanomaterial platforms for drug delivery: features, therapeutic advantages, and clinical/marketed status.

Nanomaterial Platform	Key Features	Therapeutic Advantages	Approved Products/Status
Liposome	Phospholipid bilayer vesicles; PEGylation to prolong circulation; size ~50–200 nm	Encapsulation of both hydrophilic & hydrophobic drugs; immune evasion; reduced systemic toxicity	**Approved products:** Doxil^®^ (doxorubicin), LipoDox^®^, AmBisome^®^ (amphotericin B) Myocet (doxorubicin-citrate) ^®^, Vyxeos(daunorubicin + Cytarabine)^®^ [7,26,27,28]
Polymeric NP/Micelle	Biodegradable polymers (PLGA, PCL); tunable size & degradation rate; surface functionalization possible	Sustained/controlled drug release; delivery of siRNA, peptides; improved bioavailability of hydrophobic drugs	**Approved/marketed:** Genexol-PM (paclitaxel micelle) [29] **Clinical trials:** NK105 and SP1049C
Dendrimer	Highly branched, monodisperse macromolecules; multivalent surface groups for drug conjugation	High drug loading; multivalent targeting; imaging + therapy (theranostics)	**Marketed:** Vivagel^®^ (SPL7013) [30]
Metallic NP	Gold, silver, iron oxide nanoparticles; unique optical/magnetic properties; possible surface modification	Theranostics; stimuli-responsive drug release (light, heat, magnetic field); antimicrobial effects	**Approved**: SPION-based MRI contrast agents (Feridex^®^, Ferumoxytol^®^) [31,32] **Clinical trials**: Photothermal therapy [33,34]
Silica NP (Mesoporous)	High surface area; tunable pore size (2–10 nm); easy surface functionalization	High drug loading; co-delivery of multiple agents; potential for imaging-guided therapy	**Preclinical studies:** Biosilica-enveloped ferritin cages [35,36]
Carbon-based NP	Diverse structural; high surface area; unique bonding properties of carbon atoms	High physical and chemical stability; tunable surface functionalization; suitable for drug/gene delivery, bioimaging, and photothermal & photodynamic therapies	**Preclinical and translational studies:** Amino acid-mimicking carbon dots for siRNA delivery [37]; nanodiamond-based treatment for apical lesions showing clinical potential [38]

NP, nanoparticle; PLGA, poly(lactic-co-glycolic acid); PCL, polycaprolactone; PEG, polyethylene glycol; e, superparamagnetic iron oxide nanoparticle; MSN, mesoporous silica nanoparticle.

**Table 2:** AI contributions across key stages of nanomaterial-based drug delivery development.

Research Stage	AI Contribution	Representative Methods	Platform/Database	Outcome
Design & Screening	Predict nanocarrier physicochemical properties	ML, ensemble models	NanoCommons	Efficient candidate ranking
Mechanistic Understanding	Reveal nonlinear synthesis–performance links	DL, multimodal integration	caNanoLab	Mechanistic insight, hypothesis generation
Optimization & Discovery	Explore novel chemical space	GAN, VAE, RL	Nanomaterial Registry	Novel design proposals with improved profiles
Clinical Translation & Regulation	Improve reproducibility and transparency	XAI, data governance	NanoSolveIT/FDA Predictive Toxicology Roadmap	Higher regulatory confidence, cost/time reduction

NanoCommons is an EU Horizon 2020 infrastructure project that standardizes nanomaterial data, ontologies, and FAIR data sharing practices for computational modeling. caNanoLab is a U.S. National Cancer Institute repository that curates nanomaterial characterization and assay data to support reproducible research. The Nanomaterial Registry maintained by the U.S. EPA aggregates physicochemical and biological attributes of nanomaterials for cross-study comparison. The NanoSolveIT project and the FDA Predictive Toxicology Roadmap provide frameworks for predictive nanotoxicology and regulatory evaluation based on AI-driven modeling.

## Data Availability

The datasets used are available on request from the authors.
